# Epidemics of England

**Published:** 1855-06

**Authors:** 


### PROGRESS OF EPIDEMICS.

### EPIDEMICS OF ENGLAND,

*For the Quarter ending March 31st,* 1855.

[From the Registrar-General's Quarterly Report.]

In the south-eastern counties the deaths were 11,253, in the place of 8446. The Epsom district suffered from scarlatina; Guildford from small-pox and measles; Farnham from fever, measles, hooping cough, and diarrhœa. The deaths, for the first time, exceed the births in Farnham. In Bexley subdistrict in Kent, there were as many as forty cases of small-pox at one time; only those unvaccinated died. Scarlatina prevailed in Folkstone, Elham, Portsmouth, the Isle of Wight, Kintbury, Farringdon, and Fyfield. In the barracks at Winchester, occupied by about 2000 men, chiefly recruits and invalid depôts, 40 men died, chiefly of acute pulmonary complaints: 46 deaths occurred in the new military hospital, Portsea. Small-pox, imported, it is believed, by the Essex rifles, was the cause of 6 deaths in Windsor, where, also, 2 children died in February of choleraic diarrhœa in a cleanly country cottage.

The south midland counties suffered from scarlatina and fever in several districts. In Oxford 25 deaths occurred from small-pox, and the deaths exceeded the births in number. In Cambridge the mortality was high. In Leighton Buzzard the deaths were nearly double the average.

In some districts of the eastern counties measles, small-pox, and hooping cough prevailed. The mortality was high in Norwich and several other places, but it was only a sixth part higher than it was in 1853. The mortality in the south-western division, where the climate is milder, rose to the same extent. In Salisbury the deaths in the winter quarters of 1853 and 1855 were 77 and 78; and the same result is noticeable in other districts, which must have been exposed to nearly the same degree of cold as the districts in which the cold winter proved most fatal. Cold is chiefly fatal to the aged and feeble, and it arrests some classes of diseases.

Influenza was epidemic in St. Agnes, Truro; typhus in Lerrin, Liskeard: Plymouth and the surrounding districts are still in an unsatisfactory sanitary state. In Bath, Clifton, and Cheltenham the mortality was above the average.

The west midland counties suffered somewhat less than the counties of the previous division. The mortality was high in Hereford, where measles was epidemic, and somewhat above the average in Gloucester, Shrewsbury, Stafford, Worcester, and Warwick: 2101 deaths were registered in Birmingham and Aston, 103 less than the deaths in the winter quarter of 1854, but 359 more than the deaths in the winter quarter of 1853.

In the north midland counties the mortality was raised to nearly the same extent (one-seventh), as in the other midland counties:

the districts of Leicester, Lincoln, Nottingham, and Derby exhibited little or no increase. Scarlatina, measles, and hooping-cough prevailed extensively in Lincolnshire.

Cheshire exhibited no increase; Lancashire only a slight increase in the mortality: 3678 deaths were registered in Liverpool and west Derby; 3262 in Manchester and Salford; the latter number being considerably above the average.

Yorkshire suffered less than the other divisions. In Leeds and Hunslet, where 1818 deaths were registered, the mortality was below the average of the place; and in Sheffield and York the mortality was about the average.

Measles and scarlatina prevailed to some extent in the northern counties, but the mortality was not raised one-thirteenth part above the average. In Monmouthshire and Wales the same diseases were epidemic, and the mortality was raised about one-sixth above the mortality of 1853.

